# Hydro-ethanolic leaf extract of *Ziziphus abyssinica* Hochst Ex A. Rich (Rhamnaceae) exhibits anti-nociceptive effects in murine models

**DOI:** 10.1186/s12906-017-1750-z

**Published:** 2017-04-26

**Authors:** Eric Boakye-Gyasi, Isaac Tabiri Henneh, Wonder Kofi Mensah Abotsi, Elvis Ofori Ameyaw, Eric Woode

**Affiliations:** 1 Department of Pharmacology, Faculty of Pharmacy and Pharmaceutical Sciences, Kwame Nkrumah University of Science and Technology, Kumasi, Ghana; 2 Department of Pharmacology, School of Medical Sciences, University of Cape Coast, Cape Coast, Ghana; 3 Department of Biomedical and Forensic Sciences, University of Cape Coast, Cape Coast, Ghana

**Keywords:** *Ziziphus Abyssinica*, Nociception, Formalin, Acetic acid, Glutamate, Carrageenan, Tail-immersion

## Abstract

**Background:**

Despite substantial advances in pain research and treatment, millions of people continue to suffer from pain and this has been attributed mainly to the unavailability of effective and safer analgesics. The use of plants as medicines is still widespread and plants constitute a large source of novel phytocompounds that might become leads for the discovery of newer, effective and safer alternatives. Various parts of *Ziziphus abyssinica* have been used in folk medicine in several African countries as painkillers. However, there is no report on the possible anti-nociceptive effects of this plant especially the leaves, hence the need for this current study.

**Methods:**

The possible anti-nociceptive activity of hydro-ethanolic leaf extract of *Ziziphus abyssinica* (EthE) was assessed in rodents using chemical (acetic acid, formalin and glutamate), thermal (tail-immersion test) and mechanical/inflammatory (carrageenan) models of nociception.

**Results:**

EthE (30-300 mg/kg, *p.o.*) dose-dependently and significantly inhibited chemical-induced nociception with a maximum inhibition of 86.29 ± 2.27%, 76.34 ± 5.67%, 84.97 ± 5.35%, and 82.81 ± 5.97% respectively for acetic acid, formalin (phase 1), formalin (phase 2) and glutamate tests at its highest dose. EthE also dose-dependently and significantly increased reaction times in both tail-immersion and carrageenan-induced hypernociceptive tests. The activities of the extract in the various models were comparable with the effect of morphine hydrochloride and diclofenac sodium used as standard analgesic drugs.

**Conclusion:**

Oral administration of hydro-ethanolic leaf extract of *Ziziphus abyssinica* ameliorates nocifensive behaviours associated with chemical-, thermal- and mechanical/inflammatory - induced nociceptive pain.

## Background

One of the most common reasons why people seek medical care is pain [1]. It is also a major reason for absenteeism from work, underemployment and unemployment and results in a huge financial loss to individuals and countries as a whole [2]. This is because aside the cost of treatment, it also complicates the treatment of other ailments [3]. Despite substantial advances in pain research and treatment, millions of people continue to suffer from pain. This has been attributed to either inappropriate use of analgesics or unavailability of effective and efficacious analgesics thereby increasing cost of pain treatment. Already, opioids and non-opioids which are mostly prescribed for the management of pain have limited use because of their numerous side effects [4]. This underscores the need to search for newer drugs with improved efficacy and safety.

The use of plants as medicines is still widespread especially in Africa [5]. Plants also constitute a large source of novel phytocompounds that might become leads for the discovery of new pharmaceutical agents which may be useful for the management and prevention of diseases and ailments [6]. One of such plants of medicinal importance is *Ziziphus abyssinica* (Hochst Ex A. Rich) which is commonly known as ‘Catch thorn’ in English and ‘Larukluror’ among the Sissala people of Ghana [7].

The leaves and other parts of the plant have been used traditionally to treat pneumonia, tonsillitis, Newcastle disease, snake bite, burns, wounds, tachycardia, pectoral pain, migraines and as pain-killers [7–10]. Extracts from different parts of the plant have been reported previously to have antioxidant [11], antimicrobial [11–13] anti-diarrheal [14] molluscicidal [15] antiplasmodial [16] and anti-ulcerogenic effects [17]. There have also been reports on the anti-nociceptive effects of various parts of plants in the *Ziziphus* genus including *Zizyphus spina-christi* [18], *Zizyphus oxyphylla* [19] and *Ziziphus mucronata* [20] in animal models. However, there is no report on the possible anti-nociceptive effects of this plant especially the leaves, hence the need for this current study. Based on the traditional use of the plant in the management of pain and previous published data on the anti-nociceptive effects of other species of Ziziphus, the present study examined the anti-nociceptive properties of the hydro-ethanolic leaf extract of *Ziziphus abyssinica* in animal models that could predict both peripheral and central-mediated pain as well as neurogenic and inflammatory pain.

## Method

### Plant collection

Fresh leaves of *Ziziphus abyssinica* were collected from Ejura (7°23′00.16″N, 1°22′00.00″W) in the Ejura-Sekyedumase Municipal of Ashanti Region in the month of October, 2015. It was authenticated by Mr. Clifford Asare of the Department of Herbal Medicine, Faculty of Pharmacy and Pharmaceutical Sciences (FPPS), Kwame Nkrumah University of Science and Technology (KNUST), Kumasi, Ghana. A voucher specimen (KNUST/HM/2016/L003) was deposited at the Department of Herbal Medicine’s herbarium.

### Plant extraction

About 1 kg of fresh matured leaves of *Ziziphus abyssinica* were air dried for fourteen days in a room and with the aid of a hammer mill pulverized into fine powder. An amount of 600 g of the powdered leaves was extracted with 4 L of 70% ^*v*^/_v_ ethanol for 48 h period using a Soxhlet apparatus (Aldrich^®^ Soxhlet Extraction Apparatus, Z556203, St. Louis, MO, USA). The extract obtained was labeled as EthE and subsequently concentrated using a rotary evaporator (Rotavapor R-215 model, BÜCHI Labortechnik AG, Flawil, Switzerland) under reduced pressure and temperature (70 °C). This was further dried on a water bath and then preserved in a desiccator containing activated silica until it was ready for use. The yield obtained was 10.8% ^*w*^/_w_.

### Phytochemical screening

Preliminary qualitative phytochemical screening was conducted on EthE using standard procedures [21].

### Animals

ICR mice (20-25 g) and Sprague-Dawley rats (170-250 g) of both sexes were bought from Noguchi Memorial Institute for Medical Research, University of Ghana, Legon, Ghana. They were kept in stainless cages (34 × 47 × 18 cm) in groups of five at the animal house facility of FPPS, KNUST, Kumasi. The animals were given normal commercial diet obtained from Agricare Limited, Kumasi, Ghana and water was given ad libitum*.* They were kept under normal laboratory conditions with regards to room temperature and humidity. In all experiments, animals were randomly assigned to either treatment or control group. All the techniques and protocols used in the study were done in accordance with established public health guidelines in “Guide for Care and Use of Laboratory Animals” [22]. Also, all protocols used in the study were approved by the Departmental Ethics Committee.

### Drugs and chemicals

The following chemicals and drugs were used in the study: carrageenan, formalin and acetic acid (British Drug House, Poole, England); morphine sulphate (Duopharma (M) Sdn Bhd, Malasia); diclofenac sodium (Denk Pharma, Germany) and L-glutamic acid (Sigma-Aldrich Inc., St. Louis, MO, USA).

### Anti-nociceptive activity

#### Acetic acid-induced writhing

The test was carried out as described previously [23, 24]. Mice (*n* = 5) received either vehicle (10 mL/kg distilled water *p.o.*), EthE (30-300 mg/kg *p.o*.) or diclofenac (10-100 mg/kg i.p.). After 60 min (*p.o*.) or 30 min (i.p.), they received intraperitoneal injection of acetic acid (10 mL/kg of 0.6% ^*v*^/_v_). They were then individually placed in testing chambers (a Perspex chamber of 15 cm × 15 cm × 15 cm). A mirror placed at 45° to the floor level allowed complete view of the animals in the camcorder (Sony-Handycam, model: HDRCX675/B, Tokyo, Japan) which was used to capture the nociceptive behaviors of the mice following acetic acid injection. This was captured for 30 min and later tracked using a public domain software JWatcher™ software Version 1.0 developed by University of California, Los Angeles, USA and Macquarie University, Sydney, Australia. The total number of writhes per every five-minute time bloc was obtained and this was used to plot time course curves from which the areas under the curves (AUCs) were calculated.

#### Formalin-induced nociception in mice

This test was performed as has been described previously [25, 26]. Seven groups (*n* = 5) of ICR mice were used for the test. Each mouse was placed in one of twenty Perspex chambers (15 cm × 15 cm × 15 cm) for 1 h prior to formalin injection for them to acclimatize to the new environment. Each group of mice received either vehicle (10 mL/kg distilled water, *p.o*.), EthE (30-300 mg/kg, *p.o*.) or morphine (1-10 mg/kg, i.p.) 60 min (*p.o*.) or 30 min (i.p.) before intraplantar injection of 5% ^*v*^/_v_ formalin (10 μL). Mice were instantly transferred into the transparent testing chamber and captured with the aid of a camcorder for 60 min as was described above under acetic acid induced writhing. A nociceptive score for every five-minute time bloc was obtained by measuring the duration and frequency of licking/biting of injected paws and the mean nociceptive score for each time bloc per five-minute determined as the product of the duration and frequency of licking/biting. The results obtained were considered as early/neurogenic phase (0 – 10 min) and late/inflammatory phase (10 – 60 min) from which time-course curves were plotted and the areas under the curve for each phase and each treatment determined and plotted.

#### Tail – Immersion test

The test was performed as previously described by Janssen [27] and Sewell and Spencer [28]. Seven groups of Sprague-Dawley rats (*n* = 5) were used. The animals were allowed to adapt to the environmental conditions in the laboratory for five days prior to the experiment. The lower 3.5 cm portion of the tail of each rat was marked and later immersed into a water bath maintained at 50 °C ± 0.5. The rats reacted within a few seconds by flicking or withdrawing their tail and this was measured with a stop watch and recorded as the reaction time. Animals were tested before and at 0.5, 1, 2, 3 and 4 h after administration of EthE (30-300 mg/kg, *p.o*.) or Morphine (1-10 mg/kg, i.p.). Animals in control group were given vehicle (10 mL/kg, *p.o*.). The cut-off time for tail immersion was 15 s to prevent tissue injuries.

The percentage maximum possible effect (%MPE) was calculated using the formula below:

$$ \% MPE=\left(\frac{L_2-{L}_1}{L_0-{L}_1}\right)\times 100 $$

Where L_1_ is the pre-drug latency, L_2_ is the post-drug latency and L_0_ is the cut-off latency.

#### Glutamate - induced hypernociception

The test was performed in mice (20 – 25 g) as previously described [29, 30]. Glutamate (20 μL; 10 μmol/paw) was administered into the ventral surface of rats’ right hind paws (*n* = 5) after they had been pretreated, 1 h with EthE (30-300 mg/kg, *p.o*.) or 30 min prior to morphine (3 mg/kg, i.p.). Animals in control group were given vehicle (10 mL/kg, *p.o*.). With the aid of a camcorder (Sony-Handycam, model: HDRCX675/B, Tokyo, Japan), the nocifensive behaviours of the mice were captured for 15 min immediately after glutamate injection and observed. The number of paw biting/licking was counted as an indication of nociception and analysed using a similar procedure described for acetic acid-induced nociception.

#### Carrageenan-induced mechanical hypernociception

Mechanical hypernociception was measured in rats as described previously [31, 32] using an analgesimeter (Model No. 15776, Ugo Basile, Comerio, Varese, Italy). The rats were trained at three different times before the day of testing and it involved gradually applying pressure to their right hind paws. The applied pressure (grams) able to elicit paw withdrawal was recorded as paw withdrawal threshold (PWT). A cut-off threshold of 250 g was set in order not to cause any injury to the paws. On the test day, animals were administered with carrageenan (100 μL of a 20 mg/mL solution) intraplantarly into the right hind paws after two baseline threshold (BT) had been taken. To establish that mechanical hypernociception has developed, PWTs were measured again at 2.5 h post carrageenan injection. Rats (*n* = 5) were then administered with either EthE (30, 100 and 300 mg/kg, *p.o.*), morphine (1, 3 and 10 mg/kg, i.p.) or diclofenac (10, 30 and 100 mg/kg, i.p.) at the third hour. Control group rats were given vehicle (10 mL/kg, *p.o*.). PWTs were taken again after every thirty minutes until the sixth hour post carrageenan injection.

Percentage maximum possible effect will be determined using the formula below:

$$ \% MPE=\left(\frac{PWT- BT}{250 g- BT}\right)\times 100 $$

### Statistical analysis

A sample size of five rats or mice per group was used in all in vivo tests. Mean ± SEM was used in presenting all data. All time-course curves in the study were analysed using two-way analysis of variance (ANOVA) with Bonferroni’s post hoc test. One-way ANOVA with Newman-Keuls’ post hoc test was used to determine differences between treatments groups (areas under curves). The equation below was used to calculate the percentage inhibition for each treatment:

$$ \% inhibition=\left(\frac{AUC_{control}-{AUC}_{treatment}}{AUC_{control}}\right)\times 100 $$

Graphpad^®^ Prism Version 7.0 (Graphpad Software, San Diego, CA, USA) for Windows was used to perform all statistical analysis with *P* < 0.05 considered statistically significant for all tests.

The dose of EthE at which 50% of the maximal response was achieved in the various tests referred to as ED_50_ was determined using a computer least squares, an iterative method involving nonlinear regression (three-parameter logistic) equation as shown below:

$$ Y=\frac{a+\left( b- a\right)}{\left(1+{10}^{\left({LogED}_{50}- X\right)}\right)} $$

Where, Y is the response starting from the bottom (a) and ending at the top (b) with X being the logarithm of dose.

## Results

### Phytochemical screening

Preliminary qualitative phytochemical screening conducted on the hydro-ethanolic extract of *Ziziphus abyssinica* leaves revealed the presence of tannins, phenols, alkaloids, triterpenes, flavonoids, phytosterols as well as reducing sugars.

### Acetic acid-induced writhing

**Fig. 1:**
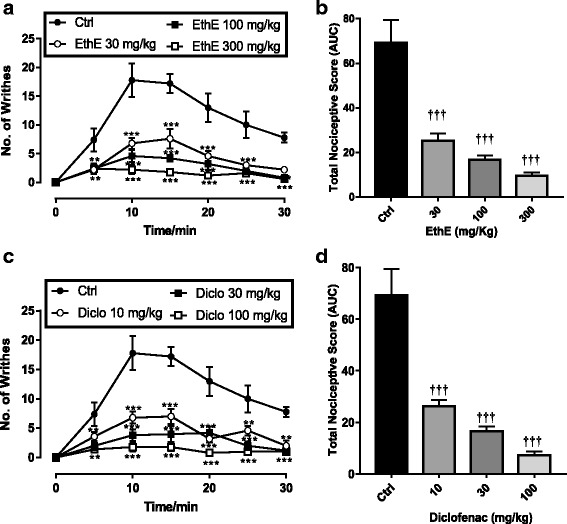
Effects of EthE (30 – 300 mg/kg, *p.o*.) and Diclofenac (10 – 100 mg/kg, i.p.) on the time-course curves (**a** and **c**) and the total nociceptive score (calculated as AUCs) (**b** and **d**) in acetic acid-induced writhing test in mice. Each data represents the mean of 5 animals and the error bars indicate S.E.M. The symbols * and † indicate significance levels compared to respective controls: ***P < 0.001 and **P < 0.01 (two – way ANOVA with Bonferroni’s post hoc). ^†††^ *P* < 0.001 (one-way ANOVA with Newman Keuls’ post hoc)

**Fig. 2:**
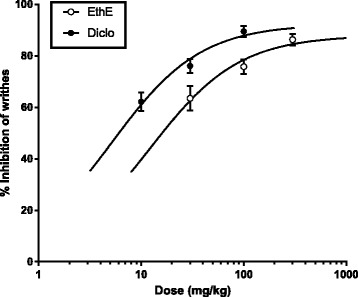
Dose-response curves of EthE (30 – 300 mg/kg, *p.o*.) and Diclofenac (10 – 100 mg/kg, i.p.) in acetic acid-induced writhing in mice

Intra-peritoneal injection of acetic acid produced abdominal writhes characterized by abdominal constrictions and stretching of at least one of the hind limbs and this was observed for a 30-min period as depicted in Fig. 1a and b. EthE significantly (F _3, 16_ = 85.49, *P* < 0.0001) and dose-dependently decreased the number of abdominal writhes with a maximum inhibitory effect of 86.29 ± 2.27% at 300 mg/kg (Fig. 1a and b). Diclofenac (10–100 mg/kg, i.p.) similarly and significantly (F _3, 16_ = 85.01, *P* < 0.0001) reduced the abdominal writhes with a maximum inhibitory effect of 89.47 ± 2.09% at 100 mg/kg (Fig. 1c and d). ED_50_ values calculated from the dose-response curves in Fig. 2 shows EthE (ED_50_:12.17 ± 1.31 mg/kg) was less potent than Diclofenac (ED_50_: 5.136 ± 1.22 mg/kg).

### Formalin-induced nociception

**Fig. 3:**
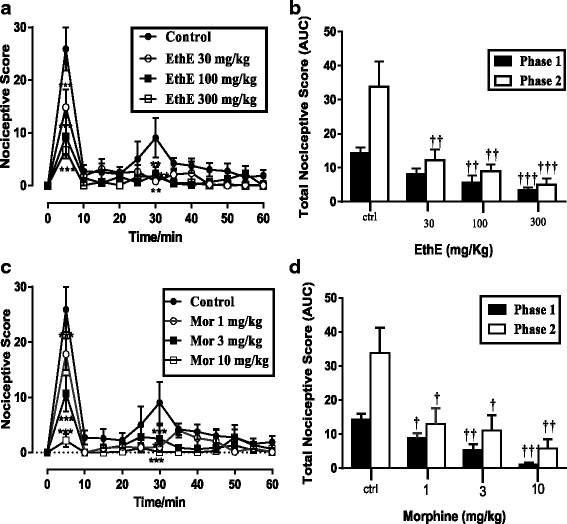
Effect of EthE (30-300 mg/kg, *p.o*.) and Morphine (1-10 mg/kg, i.p.) on the time-course curves (**a** and **c**) and the total nociceptive score (calculated as AUCs) (**b** and **d**) in formalin-induced nociceptive test in mice. Each data represents the mean of 5 animals and the error bars indicate S.E.M. The symbols * and † indicate significance levels compared to respective controls: ***P < 0.001, **P < 0.01 and *P ≤ 0.05 (two – way ANOVA followed by Bonferroni’s post hoc). †††*P* < 0.001 ††*P* < 0.01 and †*P* ≤ 0.05 (one-way ANOVA followed by Newman Keuls’ post hoc)

Injection of formalin (5%, 10 μL) intraplantarly into the right hind paws of mice resulted in a distinctive biphasic reaction – an immediate and intense pain response in the first/neurogenic phase (0–10 min) followed by a late/inflammatory phase (10–60 min) pain response which started slowly but persisted longer as shown in Fig. 3a and c. EthE (30–300 mg/kg) administered orally 1 h prior to formalin injection in a significant (*P* < 0.0001, Fig. 3a and b) and dose-dependent manner reduced nociceptive responses in both phases of the formalin test. The highest dose used produced a maximum inhibition of 76.34 ± 5.67% and 84.97 ± 5.35% respectively in the neurogenic and inflammatory phases (Fig 3b). Morphine (1–10 mg/kg, i.p.) similarly and significantly (*P* < 0.0001, Fig. 3c and d) reduced both phases of pain response with a maximum inhibition of 82.66 ± 8.11% and 95.91 ± 2.67% respectively in the neurogenic and inflammatory phases.

**Fig. 4:**
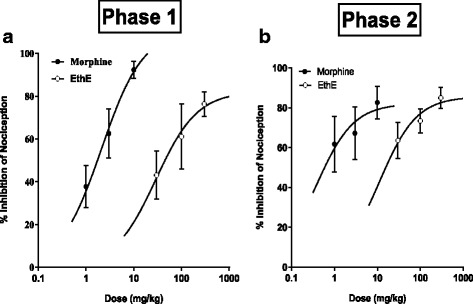
Dose response curves of EthE (30-300 mg/kg, *p.o*.) and Morphine (1-10 mg/kg, i.p.) in **a** Phase 1 and **b** phase 2 of formalin-induced nociception in mice

Dose-response curves were obtained by non-linear regression as shown in Fig. 4 and the ED_50_ values calculated for EthE by non-linear regression from the dose-response curve were 11.13 ± 1.79 mg/kg and 82.17 ± 2.10 mg/kg for phase 1 and phase 2 respectively. However, morphine was more potent with ED_50_ of 0.38 ± 2.81 and 2.11 ± 0.37 mg/kg for phase 1 and phase 2 respectively.

### Tail-immersion test

**Fig. 5:**
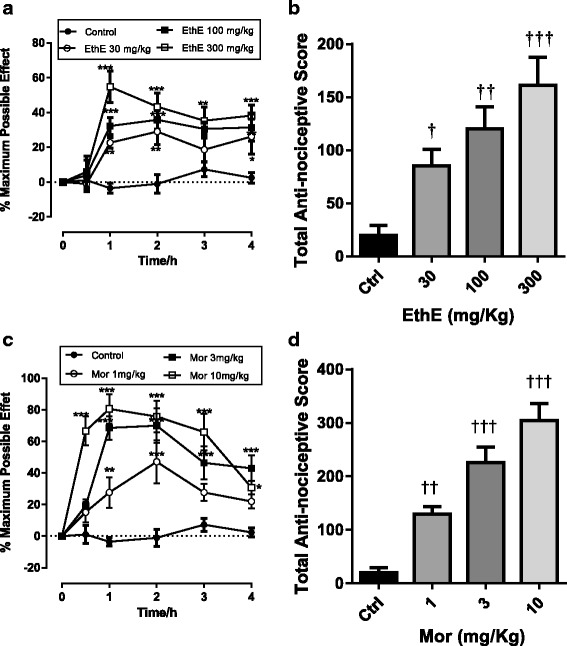
Effect of EthE (30 – 300 mg/kg, *p.o*.) and morphine (1 – 10 mg/kg, i.p.) on the time-course curves (**a** and **c**) and the total nociceptive score (calculated as AUCs) (**b** and **d**) of tail-immersion test in rats. Each data represents the mean of 5 animals and the error bars indicate S.E.M. The symbols * and † indicate significance levels compared to respective controls: ***P < 0.001, **P < 0.01 and *P < 0.05 (two – way ANOVA followed by Bonferroni’s post hoc). †††*P* < 0.001, ††*P* < 0.01 and †*P* < 0.05 (one-way ANOVA followed by Newman-Keuls’ post hoc)

**Fig. 6:**
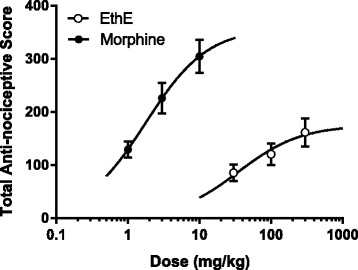
Dose-response curves of EthE (30-300 mg/kg, p.o.) and morphine (1-10 mg/kg, i.p.) in tail-immersion test in rats

Effects of various drug treatments calculated as a percentage of the maximum possible effect (% MPE) were used to plot the time-course curves which revealed a marked effect on the tail withdrawal latencies of rats used as shown in Fig. 5a and c. EthE (30-300 mg/kg, *p.o*.) significantly (F _3, 16_ = 9.817, *P* = 0.0007) and dose-dependently increased tail withdrawal latencies of rats to a total anti-nociceptive score of 161.1 ± 26.54 at the highest dose used (Fig. 5b). Morphine (1-10 mg/kg, i.p.) also significantly (F _3, 16_ = 28.46, *P* < 0.0001) increased tail withdrawal latencies of rats with a total anti-nociceptive score of 304.4 ± 31.54 as shown on Fig. 5d. The ED_50_ values calculated by non-linear regression analysis were 35.25 ± 1.92 mg/kg and 1.77 ± 1.48 mg/kg for EthE and morphine respectively with the latter being more potent and more efficacious as shown in Fig. 6 with the differences in their maximal responses.

### Glutamate-induced hypernociception

**Fig. 7:**
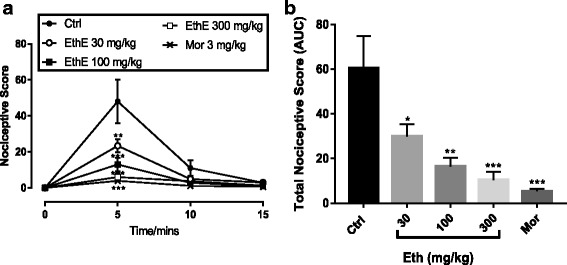
Effect of EthE (30 - 300 mg/kg, *p.o.*) and morphine (3 mg/kg, i.p.) on (**a**) the time course curve of glutamate-induced neurogenic pain and the AUC (**b**). Each data represents the mean of 5 animals and the error bars indicate S.E.M. The symbols * and † indicate the significance levels compared to respective controls: ***P < 0.001; (two-way ANOVA followed by Bonferroni’s post hoc test); †††*P <* 0.001, ††*P <* 0.01, †*P <* 0.05, (one-way ANOVA followed by Newman-Keuls’ post hoc test)

EthE (30–300 mg/kg, *p.o*.) and morphine (3 mg/kg, i.p.), during the fifteen (15) minutes observational period, significantly (*F*_*4, 77*_ *=* 11.02*, P < 0.0001,* Fig. 7a) reduced nociceptive behaviours of mice which was characterized by flinching, licking and biting of paws following intraplantar injection of glutamate (20 μL; 10 μmol/paw). The total nociceptive effect of glutamate was significantly (*F*_4, 20_ = 9.419; *P* < 0.0002, Fig. 7b) reduced by both EthE and Morphine with a maximal inhibition of 82.81 ± 5.97% and 91.12 ± 1.83% respectively.

### Carrageenan-induced mechanical hypernociception in rats

**Fig. 8:**
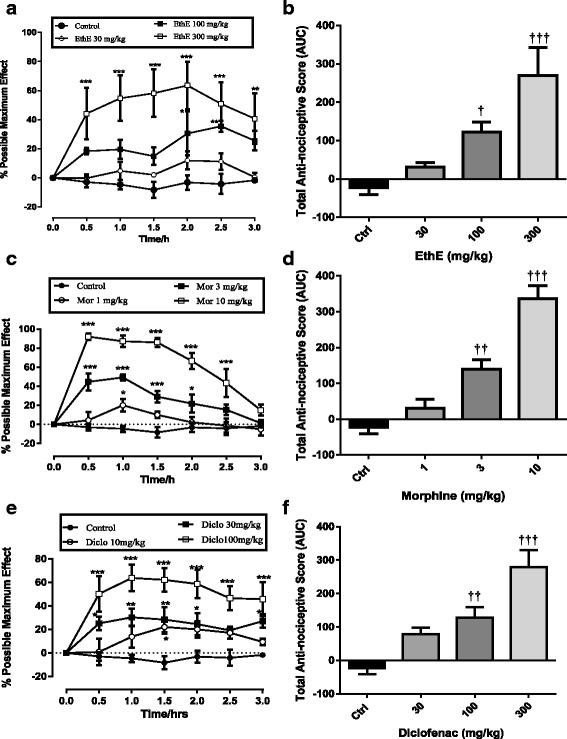
Effect of EthE (30-300 mg/kg, *p.o*.), morphine (1-10 mg/kg, i.p.) and diclofenac (10-100 mg/kg, i.p.) on time course curves (**a**, **c** and **e**) of carrageenan-induced mechanical hypernociception in rats and total nociceptive score (**b**, **d** and **f**) (calculated as AUC). Each data represents the mean of 5 animals and the error bars indicate S.E.M. The symbols * and † indicate the significance levels compared to respective controls: ***P < 0.001, **P < 0.01 *P < 0.05 (two – way ANOVA followed by Bonferroni’s post hoc). †††*P* < 0.001, ††*P* < 0.01 and †*P* < 0.05 (one-way ANOVA followed by Newman-Keuls’ post hoc)

Rats showed baseline withdrawal thresholds of about 70 to 130 g on the day of experiment. Two and a half hours (2.5 h) after carrageenan injection, the ipsilateral paws showed a marked mechanical hypernociception in all rats used which was maintained in vehicle-treated animals throughout the entire duration of the test. A change in paw withdrawal threshold was calculated as a percentage of the maximum possible effect (% MPE). EthE (30–300 mg/kg *p.o*.) administered 3 h post carrageenan injection produced a significant (F _3, 16_ = 10.29, *P* = 0.0005) and a dose-dependent reversal of mechanical hypernociception (Fig. 8a). The highest dose of EthE markedly reversed the carrageenan-induced mechanical hypernociception with a mean total anti-nociceptive effect of 269.9 ± 72.41 as shown in Fig. 8b. Morphine (1-10 mg/kg) after intraperitoneal injection similarly reversed mechanical hypernociception significantly (F _3, 16_ = 34.4, *P* < 0.0001) in a dose-dependent manner as shown in Fig. 8c; with the highest dose completely reversing the hypernociception with an average total nociceptive score of 336.9 ± 35.19 as shown in Fig. 8d. Also, the intraperitoneal injection of diclofenac (10–100 mg/kg) significantly (F _3, 16_ = 14.77, *P* < 0.0001) and dose dependently relieved the mechanical hypernociception as depicted in (Fig. 8e). The highest dose of diclofenac also completely reversed the carrageenan-induced mechanical hypernociception with an average total anti-nociceptive score of 279.1 ± 50.85 (Fig. 8f).

**Fig. 9:**
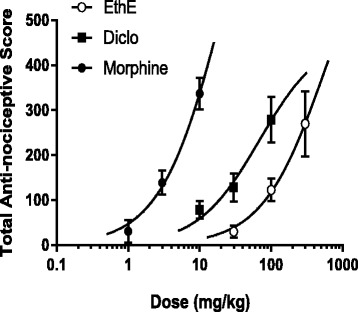
Dose-response curves of EthE (30-300 mg/kg, *p.o*.), morphine (1-10 mg/kg, i.p.) and diclofenac (10-100 mg/kg, i.p.) in carrageenan-induced mechanical hypernociception in rats

ED_50_ values calculated by non-linear regression were 21.29 ± 2.94 mg/kg, 69.01 ± 2.26 mg/kg and 554 ± 6.12 mg/kg for Morphine, diclofenac and EthE respectively as shown on Fig. 9.

## Discussion

Preliminary qualitative phytochemical screening on the extract revealed the presence of tannins, reducing sugars, phenols, alkaloids, triterpenes, phytosterols and flavonoids. The results confirm the assertion made by Tiwari et al. [21] that ethanol has the ability to pull out these phytochemicals when used as the solvent for extraction. Secondary plant metabolites contain the chemical compounds responsible for the healing properties of medicinal plants as they produce specific pharmacological effects in humans and animals [33]. Importantly, the presence of these biologically active phytochemicals has been reported by several researchers to account for the therapeutic effects of many medicinal plants [34–36]. For instance, flavonoids are reported to have analgesic and anti-inflammatory effects and the mechanism by which they exhibit these effects have been proposed to involve the inhibition of cyclo-oxygenase (COX) and lipoxygenase effects, neutrophil degranulation and eicosanoid biosynthesis [37–39]. Also, several alkaloids have been reported to contribute to analgesic activities of medicinal plants [40]. The use of *Z. abyssinica* in traditional medicine may therefore be validated by the presence of these phytochemicals of known health benefits and as such further pharmacological studies on the species are needed.

Anti-nociceptive effect of EthE was first of all evaluated in acetic acid - induced abdominal writhing model in mice as this model is reliable, simple, sensitive and particularly suitable for evaluating even weaker analgesics [41]. Intraperitoneal administration of acetic acid is known to trigger the synthesis and / or release of prostaglandins which subsequently cause the production of bradykinin, a noxious endogenous substance within the peritoneum resulting in abdominal writhing [42, 43]. Other related studies have also implicated interleukins IL-1β, IL-8 and tumour necrosis factor –alpha (TNF-α) from mast cells and resident macrophages within the peritoneum [44]. Data obtained from this study indicated that EthE prominently inhibited acetic acid-induced nociception. This may imply that EthE inhibited the release and / or synthesis of inflammatory mediators and pro-inflammatory cytokines. It is also possible that EthE partially or completely blocked the receptors to these mediators.

Though acetic acid-induced writhing test can detect both weak and strong analgesics, it equally detects some non-analgesics such as antihistamines, muscle relaxants, monoamine oxidase inhibitors among others [41, 45] and this has been an important limitation of the test. Therefore, in an attempt to avoid false positive results, formalin test was used to further confirm the analgesic effect of the extract. Formalin test is particularly very useful for the evaluation of new analgesics since it encompasses neurogenic, inflammatory and central mechanisms of nociception [46, 47]. It is also very predictive and a valid model for mimicking acute and clinical pain [41]. A distinct biphasic pain reaction usually results when 5% formalin is injected intraplantarly into mice hind paws. This is characterized by an immediate acute pain occurring within the first ten (10) minutes after formalin injection and a late phase starting between 10 and 15 min and lasting for about 45 min [47, 48]. The first phase of pain induction by formalin is as a result of direct activation of pain receptors whereas the second phase of pain response appears to be due to inflammatory processes in the tissues within the periphery and some significant alterations in the neurons within dorsal horn of the central nervous system which become sensitized to injurious stimuli [48, 49]. Morphine is known to inhibit both phases whereas NSAIDs mostly affect the inflammatory phase [41]. The extract, EthE exhibited an obvious anti-nociceptive activity in all phases of the test. This implies EthE may have a direct effect on pain mediators such as substance P and bradykinin or on pain receptors associated with the early phase of the test. Additionally, the inhibition of pain in the second phase may be due to a modulatory effect on the release and/ or synthesis of inflammatory and pro-inflammatory mediators through peripheral and/ or central mechanisms.

To further investigate the involvement of central pain pathways in the anti-nociceptive effect of EthE, tail-immersion test was used. Substances showing significant analgesic activity in tail-immersion test implicate both spinal and supra-spinal analgesic pathways as this test was developed to detect such compounds [50, 51]. Aside the peripheral mechanisms involving the inhibition of the release of prostaglandins, leukotrienes, bradykinins and other endogenous substances, nociception can be modulated centrally through complex processes involving the opioidergic, serotoninergic, dopaminergic and adrenergic mechanisms [52–55]. Pain threshold in the test was significantly increased by EthE and this suggests that it may be acting spinally or supra spinally to interfere with the nociception process.

EthE also elicited a prominent amelioration in glutamate-induced nociception in mice. Peripheral, spinal and supraspinal pathways of nociception involving the activation of glutamate receptors - kainate, N-Methyl-D-aspartate (NMDA) and α-amino-3-hydroxy-5-methyl-4-isoxazolepropionic acid (AMPA) receptors - are known to play a vital role in glutamate-induced nocifensive behaviours [30, 56, 57]. Glutamate plays major role in pain perceptions by acting through peripheral, spinal, and supraspinal sites of actions using both N-methyl-D-aspartate (NMDA) and non-NMDA receptors. Additionally, glutamate is also reported to induce the synthesis and release of several proinflammatory mediators including nitric oxide (NO) and NO-related and arachidonic acid-related substances in both central and peripheral nervous systems [58]. The inhibitory effects of the extract on glutamate-induced nociception might be due the extract being able to interfere with the pain perception effects of glutamate either at the periphery, spinal or supraspinal sites.

To further confirm the analgesic activity of EthE, carrageenan-induced mechanical hypernociception model was employed. In this model, inflammatory pain was induced by intraplantar injection of carrageenan. Both central and peripheral nociceptive pathways have been implicated in inflammatory pain. Nociceptors within the periphery become sensitized leading to important central changes resulting in central sensitization and hypersensitivity [29]. As such, slowly-adapting mechanoreceptors which are primarily C-fibers situated in subcutaneous and cutaneous tissues of the inflamed hind paws are easily activated by the application of subthreshold mechanical stimulus [59, 60]. However this state of hypernociception was reversed dose-dependently by EthE, morphine and diclofenac suggesting they may have peripheral and/or central mechanisms involve in the anti-nociceptive effects of EthE.

## Conclusion

In conclusion, oral administration of a hydro-ethanolic leaf extract of *Ziziphus abyssinica* ameliorates pain-related behavior in chemical, thermal and mechanical/inflammatory-induced nociceptive murine pain models with possible peripheral, spinal and or supraspinal mechanisms which could explain the usefulness of this plant as a pain remedy in traditional medicine.

## Abbreviations

AMPA: α-amino-3-hydroxy-5-methyl-4-isoxazolepropionic acid
ANOVA: Analysis of variance
AUCs: Areas under the curves
BT: Baseline threshold
COX: Cyclo-oxygenase
EthE: Hydro-ethanolic extract of *Ziziphus abyssinica*
FPPS: Faculty of Pharmacy and Pharmaceutical Sciences
IL: Interleukin
KNUST: Kwame Nkrumah University of Science and Technology
NMDA: N-Methyl-D-aspartate
NSAIDs: Non-steroidal anti-inflammatory drugs
PWT: Paw withdrawal threshold
SEM: Standard error of mean
TNF-α: tumour necrosis factor alpha
